# Recommendations for influenza and *Streptococcus pneumoniae* vaccination in elderly people in China

**DOI:** 10.1002/agm2.12102

**Published:** 2020-03-18

**Authors:** Qiong Chen, Lijing Wang, Mingxuan Xie, Xiaoying Li, Recommendations for Influenza, Streptococcus pneumoniae Vaccination in Elderly People in China Writing Group, Geriatric Respiratory Group and Chinese Society of Geriatrics

**Affiliations:** ^1^ Department of Geriatrics Department of Respiratory Medicine Xiangya Hospital Central South University Changsha China; ^2^ National Clinical Research Center for Geriatric Disorders Xiangya Hospital Central South University Changsha China; ^3^ Department of Cardiovascular Medicine Chinese PLA General Hospital Beijing China

**Keywords:** elderly people, influenza, *Streptococcus pneumoniae*, vaccination

## Abstract

Influenza and pneumonia can be prevented by vaccination, but they remain major causes of morbidity and mortality in age‐related diseases. In most areas of China, the rates of influenza and pneumococcal vaccination are relatively low and public awareness of vaccination remains insufficient. Thus, it is essential to recommend influenza and *Streptococcus pneumoniae* vaccination to elderly people in clinical practice. Based on recently published studies and related documents issued by several vaccination authorities, such as the World Health Organization, the National Health and Wellness Committee, the Chinese Center for Disease Control and Prevention, the US Centers for Disease Control and Prevention, and the US Advisory Committee on Immunization Practices, we propose official recommendations for influenza and *S pneumoniae* vaccination in elderly people in China.

## 1 INTRODUCTION

Although they can be prevented by vaccination, influenza and pneumonia are major causes of morbidity and mortality in age‐related disease. In most areas of China, the influenza and pneumococcal vaccination rates are relatively low and public awareness of vaccination is insufficient. Therefore, there is a pressing need for raising public awareness of vaccination and immunization coverage rates. Public awareness of vaccination is closely related to popularization of scientific knowledge and health education by medical personnel. To ensure public awareness, medical staff—particularly clinicians—should make systematic and scientific efforts to popularize knowledge of influenza and *Streptococcus pneumonia* vaccination and aim towards standardized vaccination recommendations and practices.

The expert consensus put forward herein is primarily based on the epidemiology and disease burden associated with influenza and pneumococcal diseases, and the evidence of vaccine safety and effectiveness. The evidence cited in this consensus report is mainly derived from relevant recently published literature and related documents issued by several vaccination authorities, including the World Health Organization (WHO), the National Health and Wellness Committee, the Chinese Center for Disease Control and Prevention, the US Centers for Disease Control and Prevention, and the US Advisory Committee on Immunization Practices (ACIP). Herein, influenza and *S pneumonia* vaccination of elderly people in China is advocated to reduce the current burden of these preventable diseases in that population.

## 2 INFLUENZA VACCINE

### 2.1 Etiology and epidemiology of influenza

Influenza viruses belong to the family *Orthomyxoviridae*. Based on their viral nuclear proteins and matrix protein antigens, they are divided into types A, B, C, and D. Type A influenza viruses are further divided into various subtypes based on differences in the surface antigens hemagglutinin (H1‐H18) and neuraminidase (N1‐N11). Type B influenza and two influenza A subtypes, A/H3N2 and A/H1N1, are considered the main pathogens responsible for human influenza epidemics.^1^, ^2^, ^3^ The epidemiological characteristics of influenza viruses include their capacity for antigenic variability and sudden outbreaks that spread rapidly, and accordingly they cause seasonal epidemics every year. Influenza outbreaks often occur in places with high population density, such as schools and nursing homes.^4^

The dominant strains and activity intensity of influenza viruses in different periods and regions exhibit distinct characteristics. Climate factors are the strongest predictors of influenza seasonality, including the lowest temperature, hours of sunshine, and maximum rainfall. Low temperature is a predictor of winter influenza occurrence and annual cyclical intensity in northern China, whereas in southern China, influenza activity is related mainly to rainfall. That knowledge can be drawn upon when devising strategies for determining the optimal times for influenza vaccination. In 2013, Yu et al^5^ demonstrated that the annual periodicity of influenza A epidemics increases with latitude, and that their spatial and seasonal characteristics are diversified. Epidemics peak in January and February in northern China, northeastern China, northwestern China, and the part of Qinghai located in the Qinghai‐Tibet area (latitude ≥ 33°N), and from April to June in southern China and Yunnan Province (latitude ≤ 27°N). Eastern China, central China, and most of the southwestern regions (latitudes 27.4‐31.3°N) experience dominant semi‐annual influenza A periodicity with peaks in January and February, and from June to August. Conversely, influenza B activity predominantly occurs in the colder months throughout most of China.

### 2.2 Disease burden of influenza in elderly people

Influenza can result in serious complications, including viral pneumonia, secondary bacterial pneumonia, encephalitis, and myocarditis, all of which can aggravate underlying medical conditions and lead to severe clinical outcomes or death.^6^ Because of factors including their reduced immune responses, lung compliance, muscle strength, and coughing reflexes, as well as a disproportionate likelihood of multiple complications and malnutrition, elderly people are a high‐risk population for influenza virus infection. Influenza infection in elderly people is associated with prolonged hospitalization and high influenza‐related mortality.^7^ Yu et al^8^ reported that influenza‐associated severe acute respiratory infection rates in adults aged ≥ 65 years in Jingzhou in Hubei Province were 141/100 000 in 2010 and 89/100 000 in 2011. Feng et al^3^ conducted a survey investigating annual influenza‐associated excess mortality in urban populations in China, which indicated that excess mortality from respiratory and circulatory diseases was 12.4 (range 7.4‐22.2) deaths per 100 000 people in the northern cities and 8.8 (range 5.5‐13.6) deaths per 100 000 people in the southern cities. Approximately 86% of deaths associated with influenza reportedly occur in people aged ≥ 65 years. The economic burden in adults aged > 60 years is significantly higher than that in those aged < 60 years, and the mean respective costs per episode in those age groups are US $2735 and $1417‐1621, respectively. In China, the influenza‐associated economic burden of inpatients aged > 60 years is 1.14 times the annual per capita income.^9^

### 2.3 Influenza vaccine development

Influenza vaccine development has involved the isolation of influenza viruses, identification of changes in circulating strains, and refinement of viral culture techniques. In 1933, scientists at the National Institute of Medical Research in London began to develop the first live attenuated influenza vaccine by successfully isolating the first influenza virus. The United States and the United Kingdom subsequently successfully developed the first monovalent type A (H1N1) inactivated influenza vaccine. Since then, influenza vaccine development has progressed remarkably rapidly. In 1942, a bivalent influenza vaccine was developed after the detection of influenza B virus. In 1978, when influenza A virus (H1N1 and H3N2) and influenza B virus were simultaneously prevalent, a trivalent influenza vaccine type A(H1N1)/A(H3N2)/B was introduced.^10^ In the late 1980s, two strains of influenza B virus were discovered, Victoria and Yamagata, which were alternately prevalent. The WHO recommended a trivalent component vaccine containing only one component from each strain, but there is no cross‐protection between the two strains, which can lead to a mismatch between the actual circulating strains and vaccine candidates for influenza B vaccine strains in component vaccines.^11^ Therefore, since 2012, recommendations issued by the WHO with regard to influenza vaccine composition have included both the Victoria and Yamagata strains, and two components that are updated annually, which constitutes a tetravalent vaccine.

Most influenza vaccines are manufactured via virus propagation in eggs or are cultured by Madin‐Darby canine kidney cells or Vero cells. Although the whole‐virus vaccine is still used in some countries, the split vaccine has become mainstream since the first split virion influenza vaccine was launched in 1968. Since then, a subunit vaccine has been obtained by purifying and removing the endoantigen on the basis of the split vaccine. To increase the immunogenicity of subunit vaccines, some manufacturers have also developed adjuvant influenza vaccines. In 2009, high‐dose inactivated influenza vaccines were first used in the United States for vaccination for adults aged 65 and over.^12^ Influenza vaccines that are currently available internationally include inactivated influenza vaccine (IIV), recombinant influenza vaccine (RIV), and live attenuated influenza vaccine, all of which are available in trivalent or tetravalent formulations. The IIVs include three types: whole‐virus vaccine, split‐virus vaccine, and subunit vaccine. The influenza vaccines currently used in China are all trivalent IIVs (TIVs).^13^

### 2.4 Influenza vaccine effectiveness and safety in elderly people

#### 2.4.1 Immunogenicity and persistence

Influenza vaccination can induce humoral and cellular immune responses in humans. In humoral immunity, influenza vaccines mainly induce the production of antibodies against influenza virus surface glycoproteins. Serum antibody levels usually peak within 2‐4 weeks after influenza virus infection or vaccination, but in elderly people it can take more than 4 weeks.^14^, ^15^ In the case of cellular immunity, CD8^+^ T lymphocytes play important roles,^16^ but both the number of T lymphocytes and their proliferative capacity decrease in elderly people. Therefore, the immune response in elderly people is comparatively lower.

Adjuvants and high‐dose influenza vaccines have been explored to improve vaccine immunogenicity. Adjuvants can enhance immune responses to influenza‐specific antigens, producing stronger immune effects than the antigens alone. Compared with a standard‐dose vaccine, high‐dose IIV3 (IIV3‐HD) improves antibody responses to influenza in adults aged 65 years or older.^17^ The protective effect of influenza vaccine lasts approximately 6‐8 months^18^ then wanes over time, and serum antibody levels are significantly reduced by 1 year after vaccination. The WHO recommends that influenza vaccine components be updated annually because of the ever‐changing antigenic nature of influenza virus. To provide maximum protection for the vaccinated population, it is still recommended that recipients vaccinate again in the current influenza season, as the antibody titers in most vaccine recipients decline substantially after a year, even if the antigenic influenza vaccine components are exactly the same as those in the previous season.^13^

#### 2.4.2 Application benefits

Vaccination is currently an effective way to prevent influenza in elderly people. In a meta‐analysis of data derived from elderly people in China, influenza vaccine had a 53% prevention effect on influenza‐like illness in recipients aged ≥60 years.^19^ Liu et al^20^ followed up 590 elderly people over the age of 60 years who received influenza vaccine in Beijing, and in that analysis, influenza vaccination was associated with reduced likelihood of developing “colds” (49.54%), “respiratory diseases” (64.54%), and “chronic diseases” (38.82%).

Vaccination against influenza can effectively reduce the number of influenza‐related emergency medical events, hospitalizations, and deaths in elderly people. Many elderly people have chronic diseases, such as diabetes, chronic obstructive pulmonary disease, coronary heart disease, and cerebrovascular disease, and influenza vaccination may have a flow‐on effect of reducing hospitalization rates and mortality rates associated with these chronic diseases in elderly people.

Vaccination is associated with a 27% reduction in the risk of hospitalization for pneumonia or influenza and a 48% reduction in the risk of death.^21^ Fireman et al^22^ examined mortality before, during, and after nine influenza seasons in relation to time‐varying vaccination status in an older population in California, United States, in which there were 115 823 deaths from 1996 to 2005, including 20 484 deaths during laboratory‐defined influenza seasons. The overall estimated vaccine effectiveness against all‐cause mortality during influenza seasons was 4.6%. Nichol et al^23^ studied 140 055 subjects in a 1998‐1999 influenza season cohort and 146 328 in a 1999‐2000 influenza season cohort, and reported that vaccination against influenza was associated with: reductions in the risks of hospitalization for cardiac disease, cerebrovascular disease (1998‐1999 season 16%, 1999‐2000 season 23%), and pneumonia or influenza (1998‐1999 season 32%, 1999‐2000 season 29%); and reductions in the risk of all‐cause mortality (1998‐1999 season 48%, 1999‐2000 season 50%).

Influenza vaccination can reduce medical expenses imposed by influenza itself and associated acute exacerbations of chronic diseases, which has significant cost effect in elderly patients with underlying diseases. In 2003, the Beijing Municipal Center for Disease Control and Prevention analyzed the efficacy and cost‐benefit analysis of influenza vaccination in 665 people aged over 60 years in Beijing and found that the cost‐benefit ratio was 1.51, which is higher than that of the general population.^24^ Approximately a third of that benefit was due to reduced onset of chronic diseases. Peasah et al^25^ performed a meta‐analysis of health economic evaluations of influenza vaccination around the world that included both high‐income and middle‐income countries, and concluded that influenza vaccination was cost‐effective. Wang et al^26^ demonstrated that influenza vaccination reduced hospitalization costs by US $1282.60 compared with patients who had not undergone influenza vaccination.

#### 2.4.3 Safety

Inoculation with IIVs is generally safe in adults.^27^, ^28^, ^29^, ^30^ The most common adverse events are transient local reactions at the injection sites, such as pain, erythema, and swelling.^13^ Vaccination recipients without a history of influenza vaccine antigen exposure may experience transient symptoms, such as fever, malaise, and myalgia,^31^ but these do not occur frequently in adults.^32^ Those who have experienced severe anaphylaxis after influenza vaccination should not seek revaccination. It has been reported that TIV recipients are more likely to develop Guillain‐Barré syndrome,^33^, ^34^ but no direct relationships between influenza vaccination and the syndrome have been reported.^35^ According to the monitoring data collated by the US vaccine adverse‐event reporting system, the total adverse reaction rate after TIV inoculation in recipients aged ≥ 65 years was approximately 16.5 per million doses from 1990 to 2005.^36^

### 2.5 Current status of influenza vaccination in elderly people in China

In China, the influenza vaccination rate remains low. Yang et al^37^ reported that the influenza vaccination rate in China’s total population was only 1.9% in 2008‐2009, and Zhou et al^38^ reported that the rate in urban residents aged > 60 years from nine cities was 4.3% in the 2011‐2012 influenza season. In efforts to increase the influenza vaccination rate, some cities (for example Zhuhai and Ningbo) now include reimbursement of influenza vaccination costs as part of medical or social insurance. Other cities, including Beijing, Karamay, Shenzhen, and Xinxiang, have implemented free vaccination policies for specific groups.^37^ Based on data from the Beijing Municipal Center for Disease Control and Prevention, after the implementation of publicly funded vaccination, the influenza vaccination rate in elderly people was approximately 50% from 2011 to 2015, which was significantly higher than it had previously been.^39^

### 2.6 Worldwide guidelines on influenza vaccination in elderly people

National influenza vaccine guidelines recommend that elderly people should receive one influenza vaccination every year.^6^, ^12^, ^40^, ^41^, ^42^ The IIV or RIV are recommended by the ACIP.^12^ The Canadian Immunization Advisory Committee recommends standard TIV or a high dose of TIV, a quadrivalent influenza vaccine, or a trivalent adjuvanted influenza vaccine.^6^ The Australian and New Zealand Geriatrics Association, the Singapore Infection Control Association, and the Infectious Diseases Association recommend one annual vaccination, but the dosage is not clearly defined.^40^, ^43^ The Korean Infectious Disease Association recommends inoculation with TIV,^42^ and the European Union Association for the Advancement of Old Medicine and the International Association of Gerontology and Geriatrics in Europe recommend standard doses of TIV or quadrivalent inactivated influenza vaccines.^41^

### 2.7 Recommendations for influenza vaccination strategies in elderly people in China

Trivalent IIVs mainly include split‐virus vaccines and subunit vaccines, and are currently approved for use in China. Quadrivalent inactivated influenza vaccines are about to be approved. Other IIVs, such as HD‐IIV and RIV, are not yet available in China. Therefore, herein only recommendations pertaining to TIV administration are provided. TIV should be administered in accordance with the relevant national regulations and vaccine instructions, such as the Regulations on the Administration of Vaccine Circulation (http://www.gov.cn/zhengce/content/2016-04/25/content_5067597.htm) and Vaccination and the Vaccination Practice (http://www.gov.cn/yjgl/2005-10/14/content_77713.htm).

#### 2.7.1 Target population and influenza vaccination schedule

Adults aged > 60 years should receive an annual TIV before the influenza season starts. To achieve better protection, elderly people should be vaccinated annually because influenza viruses are prone to rapid antigenic mutation. When vaccine supply is limited, priority should be given to: adults with cardiovascular (excluding isolated hypertension), chronic pulmonary, hepatic, renal, hematologic, neurologic, neuromuscular, or metabolic disorders; adults who are immunocompromised; and medical staff working in health institutions, nursing homes, and sanitariums. Vaccinating health‐care workers against influenza not only protects them and keeps health services running during the influenza season, it also reduces the chances of them transmitting the virus to people at high risk.

#### 2.7.2 Inoculation method

The standard vaccination procedure is one dose contained in 0.5 mL injected intramuscularly. The deltoid muscle of the upper arm is the preferred administration site. In patients with thrombocytopenia or other hemorrhagic disorders who are at a comparatively high risk of bleeding after receiving an intramuscular injection, a deep subcutaneous injection can be administered.

#### 2.7.3 Contraindications

People who are allergic to any active components, adjuvants, or trace ingredients in influenza vaccines, such as eggs (ovalbumin or chicken protein), neomycin, formaldehyde, or Triton X‐100, should not be vaccinated.

#### 2.7.4 Adverse effects and management

The common adverse effects are local and systemic reactions. The local reactions include redness and swelling, pain, bruising, and induration of the injection site.^13^ Systemic effects include fever, chills, headache, sweating, myalgia, joint pain, discomfort, and fatigue.^31^ Those reactions generally do not require treatment and resolve spontaneously within 2 days. Rarer adverse effects include the development of a severe febrile reaction (body temperature > 39°C), which can be treated with natural cooling or medication. Some recipients may experience urticaria after vaccination. A very small minority of recipients reportedly develop anaphylactoid purpura. Such reactions should be addressed immediately via anti‐allergy treatment, such as the administration of glucocorticoids. Anaphylactic shock is extremely rare, but if it occurs it must be managed actively in accordance with routine anaphylactic shock treatment principles.

#### 2.7.5 Warnings and precautions

People should inform the doctor of any immunodeficiency conditions they have or immunosuppressant medications they are taking before influenza vaccination. To prevent allergic reactions after vaccination, appropriate monitoring and emergency rescue measures should be prepared in advance. Vaccination can prevent infection by influenza strains contained in the vaccine from 2 to 3 weeks after vaccination. Notably, however, people may still develop influenza if they are exposed to the virus before or immediately after vaccination because the influenza incubation period is typically several days. Different injection sites should be used when influenza vaccination is administered in conjunction with other vaccines. Vaccination should be postponed if the intended recipient has a fever, acute infection, or an acute attack of chronic disease. Influenza vaccination should also be administered with caution in patients who have developed Guillain‐Barré syndrome within 6 weeks of a previous influenza vaccination. All vaccine recipients should be kept under observation for 30 minutes after immunization.

## 3 PNEUMOCOCCAL VACCINE

### 3.1 Etiology and epidemiology of pneumococcal disease

*Streptococcus pneumoniae*, also known as pneumococcus, is a gram‐positive encapsulated diplococcus bacterium. It is ubiquitous in the natural environment and humans are its primary natural host. The polysaccharide capsule of the bacterium is an essential virulence factor, and more than 90 serotypes have been defined based on differences in capsule compositions. The serotype spectrums vary in different age groups and different seasons and regions, but the general distributions of the most common pathogenic serotypes are the same throughout the world.^44^

The transmission of *S pneumoniae* mainly occurs via direct respiratory droplets or infection caused by bacterial colonization in the respiratory tract. It spreads locally to the sinuses and middle ear, or is inhaled into the lower respiratory tract, which can lead to pneumonia. Invasive pneumococcal diseases (IPDs), such as meningitis, streptococcal pneumonia, and bacteremia, occur when the bacteria invade the bloodstream; these diseases are associated with high mortality. *Streptococcus pneumoniae* results in bacteremia pneumonia in approximately 20% of cases. Reportedly, up to 30 *S pneumoniae* serotypes are responsible for more than 80% of IPDs in all age groups globally. Although IPD cases are not as common as non‐IPD infections, an etiological diagnosis is more accessible in cases of IPD. Accordingly, the incidence of IPD is often considered an important indicator of *S pneumoniae* serotype distribution and disease burden.^44^

### 3.2 Pneumococcal disease burden in elderly people

Pneumococcal infection is a major cause of morbidity and mortality in elderly people. In developed countries, the annual incidence rate of IPD is approximately 8‐34/100 000, whereas in people aged ≥65 years it is approximately 24‐85/100 000.^45^ The mortality rates of pneumococcal bacteremia are reportedly 16%‐36% in all adults^46^ and 20%‐40% in older patients.^47^ In China, although the proportions of pneumococcal infections in cases of community‐acquired pneumonia (CAP) reportedly differ in different regions, varying from 28.0% to 71.5%,^48^, ^49^, ^50^ *S pneumoniae* is always the most common cause of CAP in elderly people.^51^

The antimicrobial resistance surveillance report on *S pneumoniae* derived from the China Antimicrobial Resistant Surveillance System states that the respective resistance rates to penicillin, cefuroxime, and erythromycin are 53.6%, 46.4%, and as high as 94.2% in elderly people aged ≥ 65 years.^52^ Pneumococcal vaccines, such as the 23‐valent pneumococcal polysaccharide vaccine (PPSV) known as PPSV23, can control the prevalence of pneumococcal diseases by reducing transmission, thereby alleviating its resistance to antibiotics, which reduces the emergence of drug‐resistant strains and prevents the resistance rate from rising.^53^ Therefore, pneumococcal vaccination is one of most effective means of reducing *S pneumoniae* drug resistance.

### 3.3 Development of pneumococcal vaccine

The first pneumococcal vaccine was a whole‐cell preparation. The first clinical trial of a pneumococcal vaccine was conducted in 1911 in native workers at gold and diamond mines in South Africa.^54^ Those vaccines were subsequently supplanted by serotype‐specific PPSVs. Hexavalent vaccine was developed and applied in clinical practice in 1946. In 1977, a 14‐valent PPSV was developed,^55^ and the aforementioned 23‐valent PPSV23 has been available since 1983.^56^ The immunogenicity of PPSV23 was weak in recipients aged < 2 years, leading to the development of pneumococcal conjugate vaccine (PCV), which is generated by combining pneumococcal capsular polysaccharide with a carrier protein. A 7‐valent PCV (PCV7) covering seven serotypes was introduced and approved by the US Food and Drug Administration (FDA) in 2000, and administered to children aged < 5 years.^57^ To cover more serotypes, PCV10 and PCV13 have been developed in the past decade. PCV10 was approved in Canada in 2008 and was approved by the European Medicines Agency in 2009. PCV13 was first approved in Chile in 2009 and was subsequently licensed by the FDA to prevent pneumococcal disease in young children.^58^ In December 2011 the FDA expanded the use of PCV13 to people aged ≥ 50 years.^59^ In September 2014, the ACIP recommended the use of PCV13 in all adults aged > 65 years.^60^ In China, the pneumococcal vaccine currently used in elderly people is PPSV23.^44^

### 3.4 Effectiveness and safety of *S pneumoniae* vaccination in elderly people

#### 3.4.1 Immunogenicity and persistence

Pneumococcal polysaccharide vaccine containing only polysaccharide antigen, which is a T cell‐independent antigen, can induce serotype‐specific immunoglobulin (Ig)A, IgM, and IgG (mainly IgG2 subclass) antibodies and promote the killing of *S pneumoniae* by leukocytes and phagocytic cells, thus inducing specific protective effects. Immune responses to capsular polysaccharide are age‐dependent and serotype‐dependent.^61^, ^62^ Antibody levels initially increase after vaccination with PPSV23 and remain elevated for at least 5 years in healthy adults, but they do decline over time.^63^ Antibody levels in elderly people tend to decline to approximately baseline level within 7 years after vaccination.^45^

Pneumococcal conjugate vaccine can elicit interaction between T‐helper lymphocytes and B lymphocytes, and induce strong immune responses and immunological memory. PCV13 can reportedly induce higher levels of antibody in elderly people than PPSV23.^60^, ^64^ Jackson et al^65^ reported that in a randomized double‐blind study involving 936 adults aged ≥ 70 years, the opsonophagocytic activity titers were significantly greater in a PCV13 inoculation group than in a PPSV23 group for 10 of the 12 serotypes common to both vaccines and serotype 6A, which is unique to PCV13. Opsonophagocytic activity responses after a follow‐on dose of PCV13 a year later demonstrated that a prior dose of PPSV23, but not PCV13, diminished the response to a subsequent administration of PCV13, which indicates better immunogenicity of PCV13.

#### 3.4.2 Application benefits

At present, PPSV23 is effective for preventing diseases associated with 23 common *S pneumoniae* serotypes, and reductions in incidence, hospitalization, and mortality rates of streptococcal diseases, such as CAP and IPD, have been documented.^44^ Xu et al^66^ investigated 600 cases aged ≥ 60 years who were divided into a PPSV23 group and a control group. In that study, vaccination with PPSV23 was associated with reduced incidence of lower respiratory tract infection (69.7%), use of antibiotics (72.6%), and hospitalization (65.9%) during the subsequent year. Subgroup analysis indicated that PPSV23 could reduce lower respiratory tract infection, antibiotic use, and hospitalization frequency in patients with chronic obstructive pulmonary disease and coronary heart diseases, and reduce lower respiratory tract infection and antibiotic use in diabetes and hypertension patients. In a prospective study of a cohort followed from 2008 to 2011 involving 27 204 individuals aged ≥ 60 years in Spain, the risk of CAP in a recently (<5 years) PPSV23‐vaccinated vaccine group was lower than that in a group that had never been vaccinated. The protective effect applied to bacteremic pneumococcal CAP, non‐bacteremic pneumococcal CAP, overall pneumococcal CAP, and all‐cause CAP.^67^ Maruyama et al^68^ reported that in a randomized double‐blind trial involving 1006 adults aged ≥ 55 years, the incidence of all‐cause pneumonia and pneumococcal pneumonia was significantly lower in a vaccinated group than in a placebo group, and mortality from pneumococcal pneumonia was significantly higher in the placebo group. In a meta‐analysis performed by Moberley et al,^69^ the PPSV23 vaccine was effective for preventing pneumonia and IPD in adults, and it was effective for preventing IPD in recipients aged ≥ 65 years. In a modeling study that added PPSV23 to the immunization schedule for elderly people in Shanghai, China, the incidence of pneumococcal disease in the PPSV23 vaccination group was lower than it was in the control group.^70^ The respective cumulative incidences of CAP, IPD, and hospitalization were reduced by 3.57%, 0.02%, and 1.06%.

The combined administration of pneumococcal polysaccharide vaccination and influenza vaccination has a partially additive benefit. A meta‐analysis on data derived from people aged ≥ 65 years reported in 2016 indicated that combined pneumococcal polysaccharide vaccination and influenza vaccination was associated with lower pneumonia and all‐cause mortality than influenza vaccination alone.^71^ Chang et al^72^ analyzed 24 429 elderly people aged ≥ 75 years in Taiwan, China, and reported that compared with influenza vaccination alone, additional pneumococcal vaccination yielded additive reductions of 26% in all‐cause mortality, 23%‐29% in hospitalization, and 6%‐13% in inpatient expenditure. Nichol^73^ followed up elderly people diagnosed with chronic lung disease for three influenza seasons. In that study, influenza vaccination alone was associated with substantial reductions in hospitalization (52%) and mortality (70%), and pneumococcal vaccination alone was also associated with reductions in hospitalization (27%) and mortality (34%)—but receiving both vaccinations resulted in partially additive benefits, including a 63% reduction in hospitalization and an 81% reduction in mortality. The results indicated that receiving both vaccines conferred additive clinical benefits in patients with chronic pulmonary diseases. In summary, previous studies have shown that simultaneous administration of influenza and pneumococcal vaccines in elderly people is effective and safe, and is not associated with increased side‐effects.^74^

Vaccinating older populations with PPSV23 is reportedly cost‐effective. Zhao et al^70^ conducted a study in people aged ≥ 60 years in Shanghai, China, in 2016 and reported that the incremental cost‐effectiveness ratio of PPSV23 vaccination compared with non‐vaccination was US $16 699/quality‐adjusted life years, which is lower than the per capita gross domestic product of Shanghai (US $16 840). In a theoretical cost‐effectiveness study conducted in Brazil by De Soarez et al^75^ and reported in 2015, a strategy involving country‐wide vaccination of all adults aged 60 years with one dose of PPSV23 avoided 7810 hospitalizations and 514 deaths, and saved 3787 years of life. The total costs of its implementation were US $31 507 012 to the health system and US $44 548 180 from a whole societal perspective. Hypothetically, the strategy resulted in respective incremental cost‐effectiveness ratios of US $1297 and $904 per life year for the health system and from the whole societal perspective, respectively, compared with current practice (vaccination of institutionalized elderly people and elderly people with underlying diseases).

#### 3.4.3 Safety

Some PPSV vaccine recipients may experience mild local reactions at the injection site, such as pain, swelling, and erythema, which generally resolve within 48 hours. In cases in which a subcutaneous injection is used or a second vaccination is performed, the aforementioned local reactions are relatively common.^76^ Serious reactions, such as local induration at the injection site and moderate systemic symptoms, such as fever or myalgia, rarely occur, and serious systemic adverse reactions, such as anaphylaxis, are very rare.^44^

### 3.5 Current status of pneumococcal vaccination in elderly people in China

Data on the epidemiological surveillance of pneumococcal vaccination in elderly people in China are limited. Data from local areas indicate that pneumococcal vaccination rates are low among elderly people. In 2010, the rate of pneumococcal vaccination in residents of the Chaoyang District of Beijing aged ≥ 60 years and living in the community was 2.1%.^77^ In 2014 that of male residents of the Qingpu District of Shanghai aged ≥ 65 years was 1.8%, and for female residents it was 2%.^78^ In the same study, the vaccination rates were 1.6% for those aged 65‐79 years, and 4.4% for those aged ≥ 80 years. In March 2015, a pneumococcal vaccination subsidy program for older residents aged ≥ 60 years was launched in Chengdu, and 1200 people were randomly selected for the survey. The vaccination rate was 42.1% in that program.^79^

### 3.6 Worldwide guidelines on pneumococcal vaccination in elderly people

Guidelines in most countries currently recommend one dose of PCV13 or PPSV23 in immunocompetent elderly people.^41^, ^42^, ^80^, ^81^, ^82^ The Australian and New Zealand Society for Geriatric Medicine, the European Union Geriatric Medicine Society, and the International Association of Gerontology and Geriatrics‐European Region all recommend PPSV23 for adults aged ≥ 65 years. Revaccination should be considered only 5‐6 years after the first vaccination, and no more than two vaccinations should be administered.^41^, ^43^ The ACIP recommends that people aged ≥ 65 years who have not previously received a pneumococcal vaccination or are not sure whether they have should be vaccinated with one dose of PCV13 first, followed by a dose of PPSV23 (PCV13‐PPSV23 sequence). The recommended interval between the PCV13 and the PPSV23 is at least 1 year for immunocompetent adults aged ≥ 65 years. Those who have chronic cardiovascular disease (excluding hypertension), chronic lung disease, chronic liver disease, diabetes, and/or alcohol or smoking habits should receive another dose of PPSV23 at least 5 years later. It is recommended that adults aged ≥ 65 years with immunocompromising conditions, functional or anatomic asplenia, cerebrospinal fluid leaks, or cochlear implants receive one dose of PCV13 first, followed by PPSV23 ≥ 8 weeks later, then another dose of PPSV23 ≥ 5 years after that. People aged ≥ 65 years who have a history of PPSV23 vaccination should receive a dose of PCV13 at least 1 year after their last dose of PPSV23.^80^

### 3.7 Recommendations for pneumococcal vaccination strategies in elderly people in China

The pneumococcal vaccine approved for use in elderly people in China is PPSV23. Although it has been circulated in China, PCV13 has not yet been approved for use in elderly people. Therefore, herein only recommendations pertaining to PPSV23 vaccination are provided. PPSV23 vaccination should follow national regulations and vaccine instructions, such as those advocated in the Regulations on Vaccine Circulation and Vaccination and Standard Practices for Vaccination.

#### 3.7.1 Target population and schedule of pneumococcal vaccine

Adults aged ≥ 60 years should receive one dose of PPSV23. Revaccination is generally not recommended in immunocompetent people, but it is now recommended that elderly people who have a high risk of severe pneumococcal infection and who have been vaccinated more than 5 years previously be revaccinated. People aged ≥ 65 years who have not been vaccinated within 5 years (including those who were aged < 65 years at the time of their last vaccination) can be revaccinated. Because data on the safety of administering PPSV23 three or more times are insufficient, additional vaccinations after the first revaccination (that is, two vaccinations in total) are not routinely recommended.

Those with the following risk factors for pneumococcal infection should be given priority: chronic illness, such as chronic pulmonary disease (eg, chronic obstructive pulmonary disease, asthma, bronchiectasis); chronic cardiovascular disease (eg, congestive heart failure or cardiomyopathies, but excluding hypertension); chronic liver disease (cirrhosis); cochlear implants; diabetes mellitus; cerebrospinal fluid leaks; alcoholism; functional or anatomic asplenia (congenital or acquired asplenia, sickle cell disease or other hemoglobinopathies, splenic dysfunction, or splenectomy); and conditions associated with reduced immune function (such as congenital or acquired immunodeficiencies, HIV, nephrotic syndrome, chronic renal failure, generalized malignancy, multiple myeloma, leukemia, lymphomas, Hodgkin’s disease, solid organ transplantation and bone marrow transplantation, and those receiving immunosuppressive medication, long‐term systemic corticosteroids, chemotherapy, or radiation therapy).^44^, ^45^, ^63^, ^83^, ^84^

#### 3.7.2 Inoculation method

The standard vaccination preparation is one dose contained in 0.5 mL, and subcutaneous or intramuscular injection is recommended. The deltoid muscle of the upper arm is the preferred administration site due to the low risk of side‐effects associated with intramuscular injection at this site.

#### 3.7.3 Contraindications

People who are allergic to any component of PPSV23 should not be vaccinated.

#### 3.7.4 Adverse reactions

The most common adverse reactions are mild to moderate fever, general body temperature ≤ 38.8°C, and local reactions, such as pain, erythema, swelling, and local indurations at the injection site. Systemic adverse reactions include weakness, fatigue, myalgia, and headache. Most of the local reactions occur within 3 days after inoculation and resolve within 5 days. The possibility of adverse reactions at the injection site after revaccination is greater than that after the first vaccination in people aged ≥ 65 years.

#### 3.7.5 Warnings and precautions

In those with severe impairment of cardiovascular and/or pulmonary function, systemic reactions at the time of vaccination may be life‐threatening, and such patients should be duly monitored. The mixing of different vaccines in the same container is forbidden, and different injection sites should be utilized for simultaneous administration of multiple vaccines. Vaccination should be delayed in people with fever, acute infection, or the acute stages of chronic disease. Finally, all vaccine recipients should be kept under observation in the inoculation observation area for 30 minutes after immunization.

## CONFLICTS OF INTEREST

This experts’ recommendations manuscript was initiated by the Special Fund for Geriatric Medicine Development of the China Health Promotion Foundation, and it was supported by Sanofi Pasteur and Merck Sharp and Dohme.

## Appendix 1

### Members of the expert group (in pinyin order of the last name)

Du Xueping (Yuetan Community Health Service Center, Fuxing Hospital, Capital Medical University); Fan Jin (Department of Geriatric Emergency, Chinese PLA General Hospital); Fang Baomin (Department of Respiratory and Critical Care, Beijing Hospital); Feng Luzhao (National Institute for Communicable Disease Control and Prevention, Chinese Center for Disease Control and Prevention); Gao Xinglin (Institute of Geriatrics, Guangdong Provincial People’s Hospital); Liu Jian (General Medical Department, Tongji Hospital Tongji Medical College of HUST); Liu Qiangui (Second Respiratory Department, Beijing Geriatric Hospital); Liu Xinmin (Department of Geriatrics, Peking University First Hospital); Sun Baojun (Department of Respiratory Medicine, South Building, Chinese PLA General Hospital); Sun Li (Department of Geriatric Respiratory Medicine, Shanxi Provincial People’s Hospital); Wang Huaqing (National Immunization Program Center, Chinese Center for Disease Control and Prevention); Wang Jianye (Department of Urology, Beijing Hospital); Wang Lin (Department of Cadre Health‐Care, Second Hospital of Tianjin Medical University); Wu Jianqing (Department of Geriatric Respiratory Medicine, Jiangsu Provincial People’s Hospital); Xu Hao (Department of Cardiology, Xiyuan Hospital CACMS); Yu Pulin (National Geriatric Medical Center, Beijing Hospital); Yu Senyang (Department of Respiratory Medicine, Chinese PLA General Hospital); Zhang Qiang (Department of Health‐Care and Medical Care, Tianjin Medical University General Hospital); Zhu Yuanjue (Department of Respiratory Medicine, Peking Union Medical College Hospital).

## References

[1] Kaji M , Watanabe A , Aizawa H . Differences in clinical features between influenza A H1N1, A H3N2, and B in adult patients. Respirology. 2003;8(2):231‐233. 12753540 10.1046/j.1440-1843.2003.00457.x

[2] Goldstein E , Cobey S , Takahashi S , Miller JC , Lipsitch M . Predicting the epidemic sizes of influenza A/H1N1, A/H3N2, and B: a statistical method. PLoS Medicine. 2011;8(7):12. 10.1371/journal.pmed.1001051 PMC3130020 21750666

[3] Feng L , Shay DK , Jiang Y , et al. Influenza‐associated mortality in temperate and subtropical Chinese cities, 2003‐2008. Bull World Health Organ. 2012;90(4):279‐288. 22511824 10.2471/BLT.11.096958 PMC3324869

[4] Pleschka S . Overview of influenza viruses In: Richt JA, Webby RJ, eds. Swine Influenza, Vol. 370 Berlin: Springer‐Verlag, Berlin; 2013:1‐20. 10.1007/82_2012_272 23124938

[5] Yu HJ , Alonso WJ , Feng LZ , et al. Characterization of regional influenza seasonality patterns in China and implications for vaccination strategies: spatio‐temporal modeling of surveillance data. PLoS Medicine. 2013;10(11):16. 10.1371/journal.pmed.1001552 PMC3864611 24348203

[6] National Advisory Committee on Immunization (NACI) . Canadian Immunization Guide Chapter on Influenza and Statement on Seasonal Influenza Vaccine for 2016‐2017. Ottawa, ON: PHAC; 2016.

[7] Ruttimann RW , Bonvehi PE , Vilar‐Compte D , Isturiz RE , Labarca JA , Vidal EI . Influenza among the elderly in the Americas: a consensus statement. Rev Panam Salud Publica. 2013;33(6):446‐452. 23939371

[8] Yu H , Huang J , Huai Y , et al. The substantial hospitalization burden of influenza in central China: surveillance for severe, acute respiratory infection, and influenza viruses, 2010‐2012. Influenza Other Respir Viruses. 2014;8(1):53‐65. 24209711 10.1111/irv.12205 PMC4177798

[9] Yang J , Jit M , Leung KS , et al. The economic burden of influenza‐associated outpatient visits and hospitalizations in China: a retrospective survey. Infect Dis Poverty. 2015;4:44. 26445412 10.1186/s40249-015-0077-6 PMC4595124

[10] Fukumi H . History of influenza vaccine. Uirusu. 1985;35(2):107‐122. 3010561 10.2222/jsv.35.107

[11] Tricco AC , Chit A , Soobiah C , et al. Comparing influenza vaccine efficacy against mismatched and matched strains: a systematic review and meta‐analysis. BMC Med. 2013;11:153. 23800265 10.1186/1741-7015-11-153 PMC3706345

[12] DiazGranados CA , Dunning AJ , Jordanov E , et al. High‐dose trivalent influenza vaccine compared to standard dose vaccine in elderly adults: Safety, immunogenicity and relative efficacy during the 2009‐2010 season. Vaccine. 2013;2012(31):861‐866. 10.1016/j.vaccine.2012.12.013 23261045

[13] Feng LZ , Yang P , Zhang T , et al. Technical guidelines for the application of seasonal influenza vaccine in China (2014‐2015). Chin J Epidemiol. 2014;35(12):1295‐1319. 25623445

[14] Cox RJ , Brokstad KA , Zuckerman MA , Wood JM , Haaheim LR , Oxford JS . An early humoral immune response in peripheral blood following parenteral inactivated influenza vaccination. Vaccine. 1994;12(11):993‐999. 7975853 10.1016/0264-410x(94)90334-4

[15] Brokstad KA , Cox RJ , Olofsson J , Jonsson R , Haaheim LR . Parenteral influenza vaccination induces a rapid systemic and local immune response. J Infect Dis. 1995;171(1):198‐203. 7798664 10.1093/infdis/171.1.198

[16] Jameson J , Cruz J , Ennis FA . Human cytotoxic T‐lymphocyte repertoire to influenza A viruses. J Virol. 1998;72(11):8682‐8689. 9765409 10.1128/jvi.72.11.8682-8689.1998 PMC110281

[17] DiazGranados CA , Dunning AJ , Kimmel M , et al. Efficacy of high‐dose versus standard‐dose influenza vaccine in older adults. N Engl J Med. 2014;371(7):635‐645. 25119609 10.1056/NEJMoa1315727

[18] Cate TR , Couch RB , Parker D , et al. Reactogenicity, immunogenicity, and antibody persistence in adults given inactivated influenza virus vaccines‐1978. Rev Infect Dis. 1983;5(4):737‐747. 6622888 10.1093/clinids/5.4.737

[19] Xin Y , Liu M . Meta analysis on the effectiveness of inactivated influenza vaccine. Chin J Epidemiol. 2009;30(4):368‐370. 19731530

[20] Liu M , Liu GF , Wang Y , et al. Study on the effectiveness and cost‐benefit of influenza vaccine elderly population in Beijing city. Chin J Epidemiol. 2005;26(6):412‐416. 16185449

[21] Nichol KL , Nordin JD , Nelson DB , Mullooly JP , Hak E . Effectiveness of influenza vaccine in the community‐dwelling elderly. N Engl J Med. 2007;357(14):1373‐1381. 17914038 10.1056/NEJMoa070844

[22] Fireman B , Lee J , Lewis N , Bembom O , van der Laan M , Baxter R . Influenza vaccination and mortality: differentiating vaccine effects from bias. Am J Epidemiol. 2009;170(5):650‐656. 19625341 10.1093/aje/kwp173 PMC2728831

[23] Nichol KL , Nordin J , Mullooly J , Lask R , Fillbrandt K , Iwane M . Influenza vaccination and reduction in hospitalizations for cardiac disease and stroke among the elderly. N Engl J Med. 2003;348(14):1322‐1332. 12672859 10.1056/NEJMoa025028

[24] Dong ZY , Wu J , Chu TX , et al. Influenza vaccine protection effect and cost‐benefit analysis. Chin J Epidemiol. 2003;24(1):80.

[25] Peasah SK , Azziz‐Baumgartner E , Breese J , Meltzer MI , Widdowson M‐A . Influenza cost and cost‐effectiveness studies globally: a review. Vaccine. 2013;31(46):5339‐5348. 24055351 10.1016/j.vaccine.2013.09.013

[26] Wang I‐K , Lin C‐L , Chang Y‐C , et al. Effectiveness of influenza vaccination in elderly diabetic patients: a retrospective cohort study. Vaccine. 2013;31(4):718‐724. 23153445 10.1016/j.vaccine.2012.11.017

[27] Margolis KL , Nichol KL , Poland GA , Pluhar RJ . Frequency of adverse reactions to influenza vaccine in the elderly: a randomized, placebo‐controlled trial. JAMA. 1990;264(9):1139‐1141. 2200894

[28] Govaert TM , Dinant GJ , Aretz K , Masurel N , Sprenger MJ , Knottnerus JA . Adverse reactions to influenza vaccine in elderly people: randomised double blind placebo controlled trial. BMJ. 1993;307(6910):988‐990. 8241913 10.1136/bmj.307.6910.988 PMC1679213

[29] Nichol KL , Margolis KL , Lind A , et al. Side effects associated with influenza vaccination in healthy working adults. A randomized, placebo‐controlled trial. Arch Intern Med. 1996;156(14):1546‐1550. 8687262

[30] Bridges CB , Thompson WW , Meltzer MI , et al. Effectiveness and cost‐benefit of influenza vaccination of healthy working adults: a randomized controlled trial. JAMA. 2000;284(13):1655‐1663. 11015795 10.1001/jama.284.13.1655

[31] World Health Organization . Vaccines against influenza: WHO position paper‐November 2012. Wkly Epidemiol Rec. 2012;87(47):461‐476. 23210147

[32] Fiore AE , Uyeki TM , Broder K , et al. Prevention and control of influenza with vaccines: recommendations of the Advisory Committee on Immunization Practices (ACIP), 2010. MMWR Recomm Rep. 2010;59(8):1‐62. 20689501

[33] Lasky T , Terracciano GJ , Magder L , et al. The Guillain‐Barré syndrome and the 1992‐1993 and 1993‐1994 influenza vaccines. N Engl J Med. 1998;339(25):1797‐1802. 9854114 10.1056/NEJM199812173392501

[34] Juurlink DN , Stukel TA , Kwong J , et al. Guillain‐Barré syndrome after influenza vaccination in adults: a population‐based study. Arch Intern Med. 2006;166(20):2217‐2221. 17101939 10.1001/archinte.166.20.2217

[35] Baxter R , Bakshi N , Fireman B , et al. Lack of association of Guillain‐Barré syndrome with vaccinations. Clin Infect Dis. 2013;57(2):197‐204. 23580737 10.1093/cid/cit222

[36] Vellozzi C , Burwen DR , Dobardzic A , Ball R , Walton K , Haber P . Safety of trivalent inactivated influenza vaccines in adults: background for pandemic influenza vaccine safety monitoring. Vaccine. 2009;27(15):2114‐2120. 19356614 10.1016/j.vaccine.2009.01.125

[37] Yang J , Atkins KE , Feng L , et al. Seasonal influenza vaccination in China: Landscape of diverse regional reimbursement policy, and budget impact analysis. Vaccine. 2016;34(47):5724‐5735. 27745951 10.1016/j.vaccine.2016.10.013

[38] Zhou L , Su Q , Xu Z , et al. Seasonal influenza vaccination coverage rate of target groups in selected cities and provinces in China by season (2009/10 to 2011/12). PLoS One. 2013;8(9):e73724. 24040041 10.1371/journal.pone.0073724 PMC3767785

[39] Zhang Y , Muscatello DJ , Wang Q , Yang P , Wu J , MacIntyre CR . Overview of influenza vaccination policy in Beijing, China: current status and future prospects. J Public Health Policy. 2017;38(3):366‐379. 28512300 10.1057/s41271-017-0079-7

[40] Hospital Influenza Workgroup Singapore . Management of novel influenza epidemics in Singapore: consensus recommendations from the Hospital Influenza Workgroup (Singapore). Singapore Med J. 2009;50(6):567‐580. 19551308

[41] Michel JP . Updated vaccine guidelines for aging and aged citizens of Europe. Expert Rev Vaccines. 2010;9(supp 3):7‐10. 20192711 10.1586/erv.10.27

[42] Choi WS , Choi J‐H , Kwon KT , et al. Revised adult immunization guideline recommended by the Korean Society of Infectious Diseases, 2014. Infect Chemother. 2015;47:68‐79. 25844267 10.3947/ic.2015.47.1.68 PMC4384453

[43] Australian and New Zealand Society for Geriatric Medicine position statement‐immunisation of older people. Australas J Ageing. 2016;35:67‐73. 27010878 10.1111/ajag.12213

[44] Chinese Prevention Medicine Association . Technical guideline on application of pneumococcal vaccine in China (2012). Chin J Epidemiol. 2012;33:1101‐1110. 23290891

[45] The World Health Organization (WHO) . 23‐valent pneumococcal polysaccharide vaccine. WHO position paper. Wkly Epidemiol Rec. 2008;83(42):373‐384. 18927997

[46] Kelley MA , Weber DJ , Gilligan P , Cohen MS . Breakthrough pneumococcal bacteremia in patients being treated with azithromycin and clarithromycin. Clin Infect Dis. 2000;31(4):1008‐1011. 11049784 10.1086/318157

[47] Evers S , Ament A , Colombo GL , et al. Cost‐effectiveness of pneumococcal vaccination for prevention of invasive pneumococcal disease in the elderly: an update for 10 Western European countries. Eur J Clin Microbiol Infect Dis. 2007;26(8):531‐540. 17570001 10.1007/s10096-007-0327-z

[48] Wang H , Yu YS , Liu Y , et al. Resistance surveillance of common community respiratory pathogens isolated in China, 2002‐2003. Chin J Tuberc Respir Dis. 2004;27(3):155‐160. 15130324

[49] Liu H , Zhang TT , Wu BY , et al. Clinical analysis of community‐acquired pneumonia in the elderly. Chin J Intern Med. 2007;46(10):810‐814. 18218228

[50] Sun HL , Xu YC , Wang H , et al. Antibiotic resistance of common pathogens isolated from community‐acquired respiratory infection in China during 2004‐2005. Chin J Infect Chemother. 2008;8(1):24‐29.

[51] Liu YN , Chen MJ , Zhao TM , et al. A multicentre study on the pathogenic agents in 665 adult patients with community‐acquired pneumonia in cities of China. Chin J Tuberc Respir Dis. 2006;29(1):3‐8. 16638292

[52] Li Y , Lv Y , Xue F , et al. Antimicrobial susceptibility surveillance of gram‐positive bacteria from Ministry of Health National Antimicrobial Resistant Investigation Net (Mohnarin) 2011‐2012. Chin J Clin Pharmacol. 2014;21(3):5133‐5137.

[53] He LJ , Xu GG , Li ZN . Research status and drug resistance mechanisms and countermeasures of *Streptococcus pneumoniae* . Geriatr Health Care. 2016;22(6):419‐421.

[54] Makela PH , Butler JC . History of pneumococcal immunization. Washington, DC: ASM Press; 2008:19‐29.

[55] Robbins JB , Austrian R , Lee C‐J , et al. Considerations for formulating the second‐generation pneumococcal capsular polysaccharide vaccine with emphasis on the cross‐reactive types within groups. J Infect Dis. 1983;148(6):1136‐1159. 6361173 10.1093/infdis/148.6.1136

[56] Song JY , Moseley MA , Burton RL , Nahm MH . Pneumococcal vaccine and opsonic pneumococcal antibody. J Infect Chemother. 2013;19(3):412‐425. 23657429 10.1007/s10156-013-0601-1 PMC3692352

[57] Pilishvili T , Lexau C , Farley M , et al. Sustained reductions in invasive pneumococcal disease in the era of conjugate vaccine. J Infect Dis. 2010;201(1):32‐41. 19947881 10.1086/648593

[58] Centers for Disease Control and Prevention (CDC) . Invasive pneumococcal disease in young children before licensure of 13‐valent pneumococcal conjugate vaccine‐United States, 2007. MMWR Morb MortaI Wkly Rep. 2010;59(9):253‐257. 20224541

[59] Food and Drug Administration . FDA expands Use of Prevnar 13 Vaccine for People Ages 50 and Older. Silver Spring, MD: US Department of Health and Human Services, Food and Drug Administration; 2011 http://www.fda.gov/newsevents/newsroom/pressannouncements/ucm285431.htm. Accessed October 10, 2012.

[60] Pilishvili T , Bennett NM . Pneumococcal disease prevention among adults: strategies for the use of pneumococcal vaccines. Am J Prev Med. 2015;49(6 suppl 4):S383‐S390. 26590438 10.1016/j.amepre.2015.09.008

[61] Simonsen V , Brandao AP , Brandileone MC , et al. Immunogenicity of a 23‐valent pneumococcal polysaccharide vaccine in Brazilian elderly. Braz J Med Biol Res. 2005;38(2):251‐260. 15785837 10.1590/s0100-879x2005000200014

[62] Lee H , Nahm MH , Kim KH . The effect of age on the response to the pneumococcal polysaccharide vaccine. BMC Infect Dis. 2010;10:60. 20219110 10.1186/1471-2334-10-60 PMC2856571

[63] Advisory Committee on Immunization Practices . Prevention of pneumococcal disease: recommendations of the Advisory Committee on Immunization Practices (ACIP). MMWR Recomm Rep. 1997;46(8):1‐24. 9132580

[64] Shiramoto M , Hanada R , Juergens C , et al. Immunogenicity and safety of the 13‐valent pneumococcal conjugate vaccine compared to the 23‐valent pneumococcal polysaccharide vaccine in elderly Japanese adults. Hum Vaccin Immunother. 2015;11(9):2198‐2206. 26176163 10.1080/21645515.2015.1030550 PMC4635730

[65] Jackson LA , Gurtman A , Rice K , et al. Immunogenicity and safety of a 13‐valent pneumococcal conjugate vaccine in adults 70 years of age and older previously vaccinated with 23 valent pneumococcal polysaccharide vaccine. Vaccine. 2013;31(35):3585‐3593. 23688527 10.1016/j.vaccine.2013.05.010

[66] Xu Y , Dong BR . Effectiveness of 23‐valent pneumococcal polysaccharide vaccine to prevent lower respiratory tract infections in the elderly population. Chin J Vaccines Immun. 2005;11(4):287‐291.

[67] Ochoa‐Gondar O , Vila‐Corcoles A , Rodriguez‐Blanco T , et al. Effectiveness of the 23‐valent pneumococcal polysaccharide vaccine against community‐acquired pneumonia in the general population aged ≥60 years: 3 years of follow‐up in the CAPAMIS study. Clin Infect Dis. 2014;58(7):909‐917. 24532544 10.1093/cid/ciu002

[68] Maruyama T , Taguchi O , Niederman MS , et al. Efficacy of 23‐valent pneumococcal vaccine in preventing pneumonia and improving survival in nursing home residents: double blind, randomised and placebo controlled trial. BMJ. 2010;340:c1004. 20211953 10.1136/bmj.c1004 PMC2834887

[69] Moberley S , Holden J , Tatham DP , Andrews RM . Vaccines for preventing pneumococcal infection in adults. Cochrane Database Syst Rev. 2013;1:Cd000422. 10.1002/14651858.CD000422.pub3 PMC7045867 23440780

[70] Zhao D , Gai Tobe R , Cui M , He J , Wu B . Cost‐effectiveness of a 23‐valent pneumococcal polysaccharide vaccine immunization programme for the elderly in Shanghai, China. Vaccine. 2016;34(50):6158‐6165. 27838068 10.1016/j.vaccine.2016.11.003

[71] Zhang YY , Tang XF , Du CH , Wang BB , Bi ZW , Dong BR . Comparison of dual influenza and pneumococcal polysaccharide vaccination with influenza vaccination alone for preventing pneumonia and reducing mortality among the elderly: a meta‐analysis. Human Vaccines Immunother. 2016;12(12):3056‐3064. 10.1080/21645515.2016.1221552 PMC5215556 27629584

[72] Chang YC , Chou YJ , Liu JY , Yeh TF , Huang N . Additive benefits of pneumococcal and influenza vaccines among elderly persons aged 75 years or older in Taiwan‐a representative population‐based comparative study. J Infect. 2012;65(3):231‐238. 22561486 10.1016/j.jinf.2012.04.014

[73] Nichol KL . The additive benefits of influenza and pneumococcal vaccinations during influenza seasons among elderly persons with chronic lung disease. Vaccine. 1999;17(suppl 1):S91‐S93. 10471189 10.1016/s0264-410x(99)00114-0

[74] Grilli G , Fuiano L , Biasio LR , et al. Simultaneous influenza and pneumococcal vaccination in elderly individuals. Eur J Epidemiol. 1997;13(3):287‐291. 9258527 10.1023/a:1007398606807

[75] De Soarez PC , Sartori AM , Freitas AC , Nishikawa AM , Novaes HM . Cost‐effectiveness analysis of universal vaccination of adults aged 60 years with 23‐valent pneumococcal polysaccharide vaccine versus current practice in Brazil. PloS One. 2015;10(6):e0130217. 26114297 10.1371/journal.pone.0130217 PMC4483239

[76] Jackson LA , Benson P , Sneller VP , et al. Safety of revaccination with pneumococcal polysaccharide vaccine. JAMA. 1999;281(3):243‐248. 9918479 10.1001/jama.281.3.243

[77] Zhang GH , Zheng DY , Shi NM , Ai X , Bai YH . Pneumonia vaccination coverage and influencing factors among part of community elderly in Chaoyang District, Beijing City, China. Chin J Biologicals. 2013;26(1):93‐95.

[78] You ZJ , Lin B , Gao Z . Pneumonia vaccination coverage and influencing factors among community elderly in Qingpu District, Shanghai City, China. Med Health Care. 2015;23(3):89.

[79] Zhu B , Huang RN , Yang RP , et al. Investigation on pneumonia vaccination coverage and influencing factors among elderly population with permanent residence of Chengdu, China. J Prev Med Inf. 2017;33(1):75‐80.

[80] Kobayashi M , Bennett NM , Gierke R , et al. Intervals between PCV13 and PPSV23 vaccines: recommendations of the Advisory Committee on Immunization Practices (ACIP). Mmwr Morb Mortal Wkly Rep. 2015;64:944‐947. 26334788 10.15585/mmwr.mm6434a4

[81] Berical AC , Harris D , Dela Cruz CS , Possick JD . Pneumococcal vaccination strategies: an update and perspective. Ann Am Thorac Soc. 2016;13:933‐944. 27088424 10.1513/AnnalsATS.201511-778FR PMC5461988

[82] Centers for Disease Control and Prevention (CDC), Advisory Committee on Immunization Practices . Updated recommendations for prevention of invasive pneumococcal disease among adults using the 23‐valent pneumococcal polysaccharide vaccine (PPSV23). Morb Mortal Wkly Rep. 2010;59(34):1102‐1106. 20814406

[83] Advisory Committee on Immunization Practices (ACIP) . Recommended adult immunization schedule: United States, 2011. Ann Intern Med. 2011;154(3):168‐173. 21282696 10.7326/0003-4819-154-3-201102010-00006

[84] Advisory Committee on Immunization Practices . Recommended adult immunization schedule: United States, 2011. Ann Intern Med. 2011;154(3):168‐173. 21282696 10.7326/0003-4819-154-3-201102010-00006

